# Successful long-term management for postoperative sternal infection with multiple disseminated lymphadenitis caused by *Mycobacterium abscessus*

**DOI:** 10.1186/s40792-023-01730-8

**Published:** 2023-08-21

**Authors:** Takahiro Yanagihara, Tomoyuki Kawamura, Kenji Minagi, Yasuharu Sekine, Kazuto Sugai, Hideo Ichimura, Yukio Sato

**Affiliations:** Department of Thoracic Surgery, University of Tsukuba Hospital, 2-1-1 Amakubo, Tsukuba, Ibaraki Japan

**Keywords:** *Mycobacterium abscessus*, Sternal infection, Disseminated infection, Mediastinitis, Surgical site infection

## Abstract

**Background:**

Postoperative sternal infection caused by *Mycobacterium abscessus* (*M. abscessus*) is rare, but associated with a high 2-year mortality rate of 40%. Decision-making around treatment strategy is challenging. Here, we present a successfully treated case of postoperative *M. abscessus* sternal infection with multiple disseminated lymphadenitis.

**Case presentation:**

The patient, an 80-year-old woman with anterior mediastinal tumor and myasthenia gravis, underwent extended thymectomy under median sternotomy. Redness appeared around the scar two months after the operation. Sternal wires were removed, debridement was performed, and the wound was kept open. *Mycobacterium abscessus* was isolated from the wound culture. The disseminated lesions in the right axillary, parasternal, and bilateral supraclavicular lymph nodes, rendered surgical options for infection control difficult; therefore, she was treated conservatively with antibiotics and negative pressure wound therapy (NPWT). The wound diminished but infectious granulation tissue remained after NPWT. Two disseminated lesions were percutaneously punctured and drained of pus, which resulted in negative cultures. Additional debridement and wound closure were performed. She was discharged after switching to oral antibiotics. No recurrence was observed five months after the antibiotics were completed (total sensitive antibiotics use: 366 days).

**Conclusions:**

Repeated culture assessment of disseminated lesions is recommended to facilitate the development of appropriate therapeutic strategies. Localized procedures may be an option for patients with controlled disseminated lesions evidenced by negative cultures.

## Background

Postoperative sternal infection caused by *Mycobacterium abscessus* (*M. abscessus*) is rare and, reportedly, associated with a high 2-year mortality rate of 40% [1]. Decision-making around treatment strategy is challenging, not only because of the treatment-resistant features of *M. abscessus* (such as high multi-drug resistance and biofilm formation), but also because disseminated lesions can form, especially in immunocompromised patients [2]. Here, we present a successfully treated case of postoperative *M. abscessus* sternal infection with multiple disseminated lymphadenitis.

## Case presentation

**Fig. 1:**
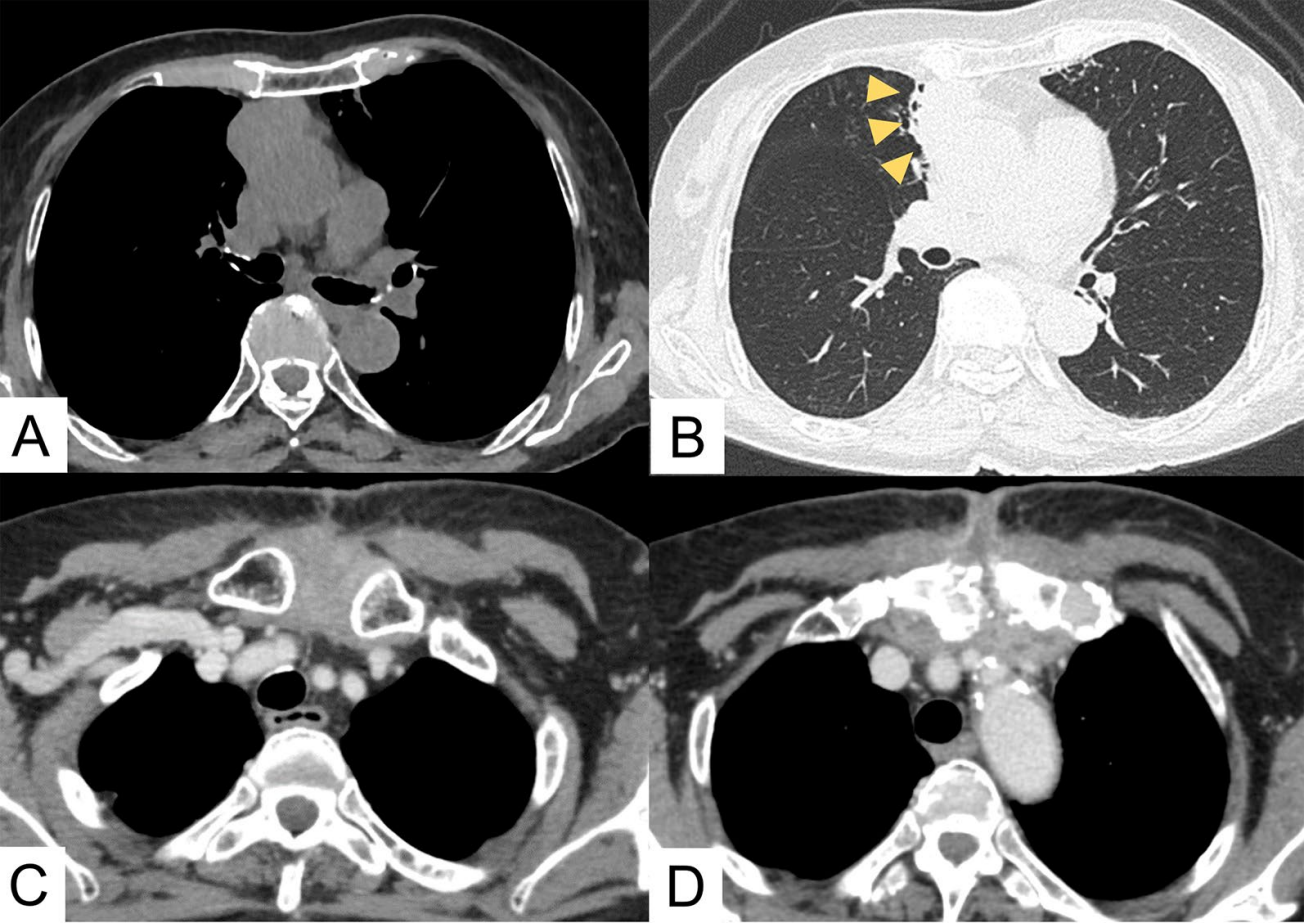
**A**, **B** Preoperative CT shows anterior mediastinal tumor adjacent to inflammatory changes in right upper lobe (yellow arrowheads). **C**, **D** CT before surgical drainage revealed abscess spreading around sternal notch to manubrium, and focal mediastinal thickening

**Fig. 2:**
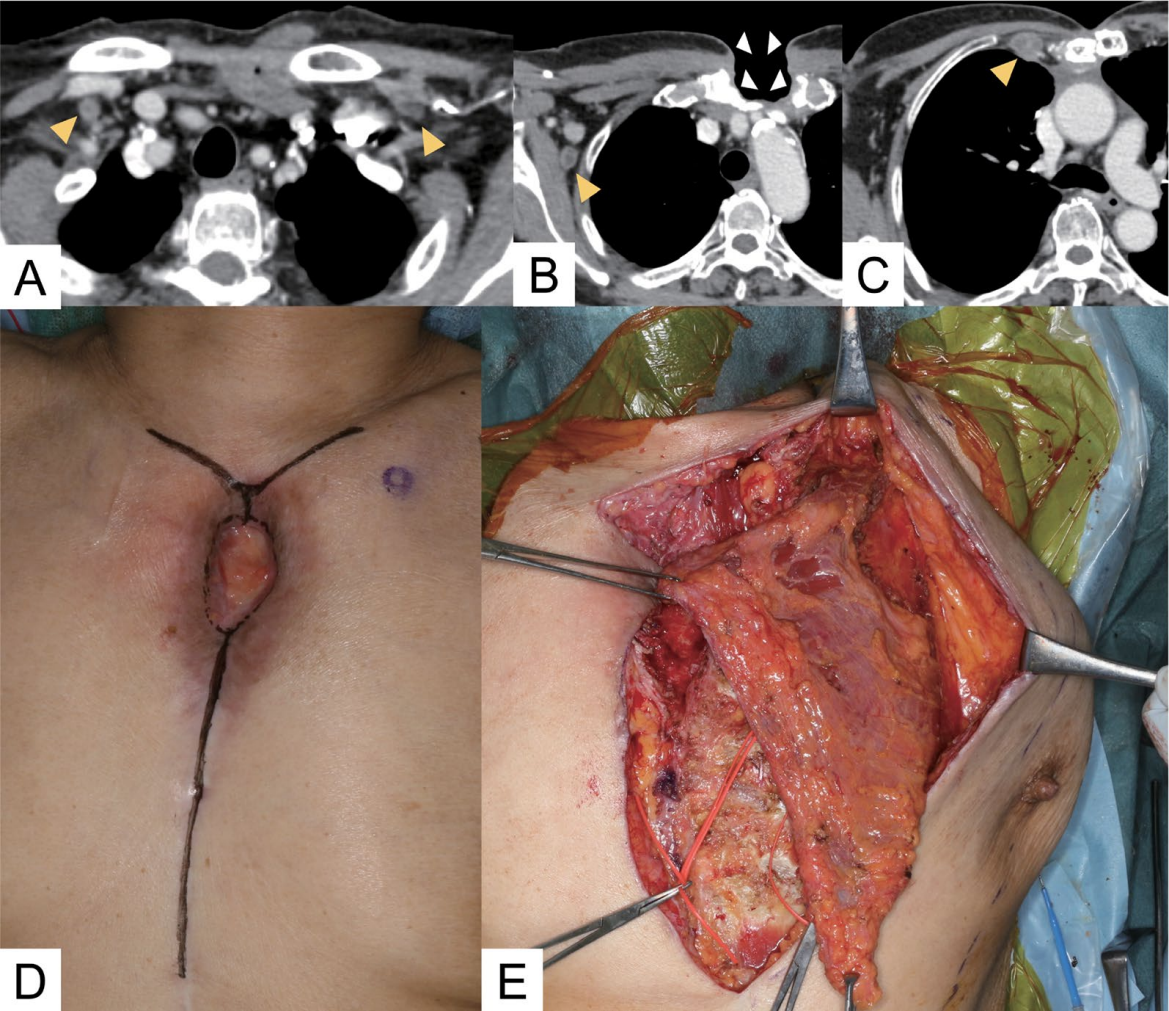
**A**–**C** CT shows lymphadenitis in bilateral supraclavicular, right axially, and parasternal lymph nodes (yellow arrowheads). The wound is left open (white arrowheads). **D** Appearance of the wound before wound closure. **E** Intraoperative findings. Left pectoralis major flap was used for wound closure to fill the space

**Fig. 3:**
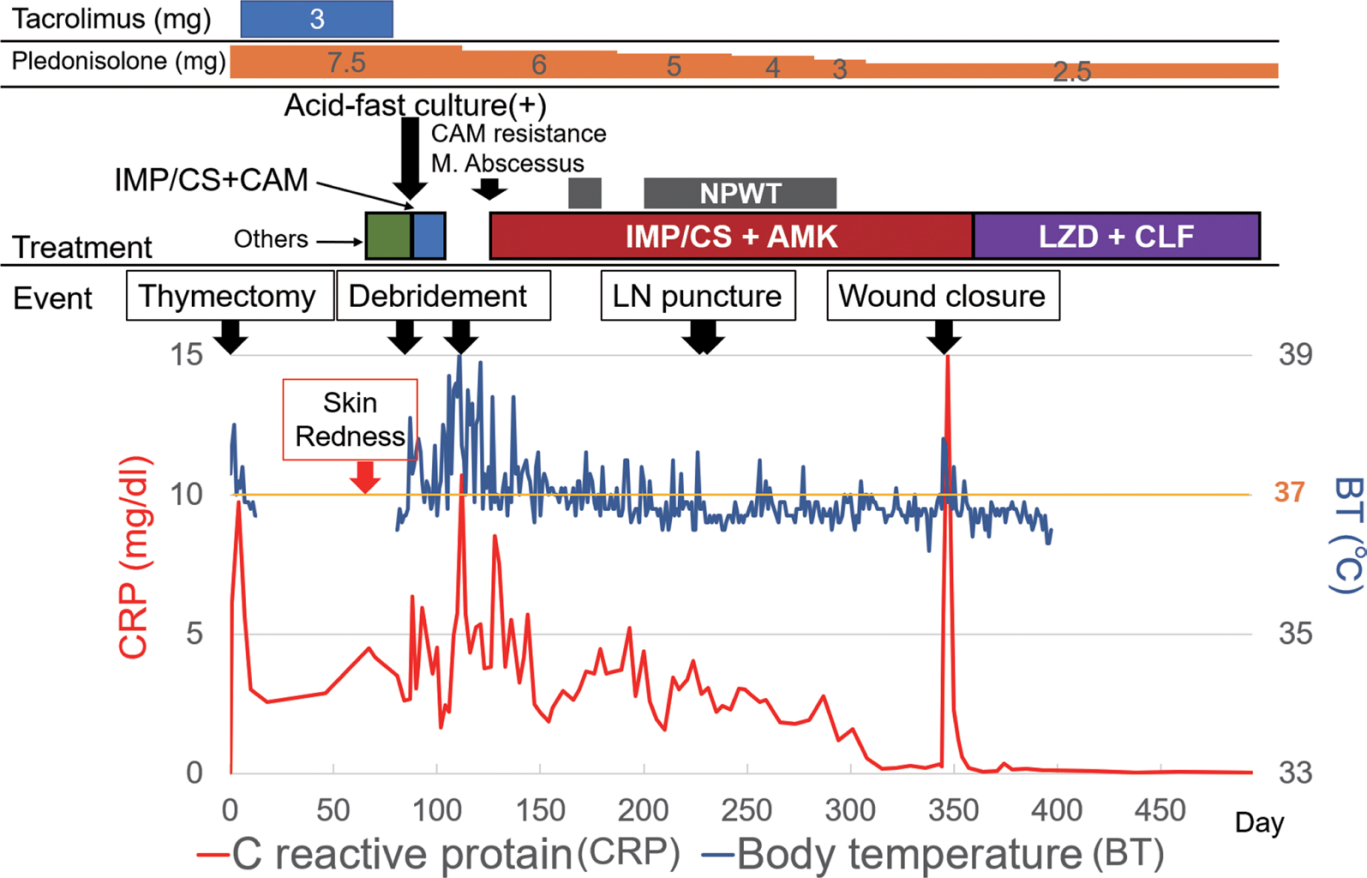
Detailed treatment course. *AMK* amikacin, *CAM* clarithromycin, *CLF* clofazimine, *IMP/CS* imipenem/cilastatin, *LN* lymph node, *LZD*, linezolid

An 80-year-woman, who lived alone, had been receiving treatment for myasthenia gravis with tacrolimus at 3.0 mg/day for 2 years. In parallel, the presence of a slowly enlarging anterior mediastinal tumor of 52 mm in diameter (craniocaudal) adjacent to inflammatory changes in the right upper lobe (Fig. 1A, B) was discovered, for which she was referred to our hospital. Positive acetylcholine receptor antibody pointed to a diagnosis of thymoma, cT1aN0M0 Stage I. Considering the patient’s age, immunodeficient status, and tumor size, we proposed two treatment options: (i) observation, and (ii) extended thymectomy under sternotomy. According to the wishes of the patient and her family, the decision was made to perform extended thymectomy. After switching from tacrolimus to prednisolone (at 7.5 mg/day), extended thymectomy via median sternotomy was performed. Partial resection of the right upper lobe was also performed, because of tumor invasion into that region of the lung. The postoperative course was uneventful. Pathological diagnosis was type B2 thymoma with tumor invasion to the right upper lobe, pT3N0M0 Stage IIIA. Adjuvant local radiotherapy was not done, in consideration of her age and social background. Two months after the operation, skin redness was first observed at the median point, cranial side, of the scar. Levofloxacin was initiated to treat the infection; however, after two weeks, pus was observed at the wound site, which was collected for general bacterial culture. She was admitted and treated with incision and drainage, and tazobactam/piperacillin with the addition of vancomycin. Seven days later, a fever occurred and computed tomography (CT) scans revealed an abscess spreading around the sternal notch to the manubrium, and focal mediastinal thickening (Fig. 1C, D). Three sternal wires and the sequestrum of the manubrium were removed. The upper side of the mediastinum was kept open under general anesthesia and, during debridement, pus was drained from the manubrium. Tissue culture from this surgery detected acid-fast bacillus; therefore, the antibiotics were changed to imipenem/cilastatin (IMP/CS) and clarithromycin (CAM). Finally, CAM was replaced with amikacin (AMK) because of the appearance of suspected drug eruption and detection of CAM-resistant *M. abscessus* in the susceptibility testing, which was proven on day 64 post-onset. Retrospective CT review indicated some inflammatory changes in the bilateral lung; therefore, bronchial lavage culture of the right upper lobe was collected at the 2nd surgery, but was negative. On day 70 post-onset, CT showed ring-enhanced low-density nodules in the bilateral supraclavicular, right axillary, and parasternal regions, indicative of disseminated lesions of *M. abscessus* infection (lymphadenitis) (Fig. 2A–C). The number and deep location of these nodules rendered complete infection control by surgical means difficult; conservative treatment was considered to be a preferable treatment strategy. Negative pressure wound therapy (NPWT) was started on day 91 post-onset. Two slightly enlarged lymph nodes were punctured and drained of pus. Long-term NPWT reduced the wound size, but the infected granulation tissue formation was prolonged after NPWT was ceased. During the treatment course (Fig. 3), the culture from the wound and C-reactive protein in the blood became negative. Moreover, the culture of pus from the two punctured lymph nodes was also negative. The decision was made to perform wound closure for the following reasons: (i) negative results of wound culture and inflammation status; (ii) the patient being unable to leave the hospital because of her inability to self-treat the wound at home; and (iii) her wish to have the wound close. Preoperative short tau inversion recovery image magnetic resource imaging (MRI) showed focal high-intensity areas in the sternoclavicular joints, suggesting residual sequestrum; therefore, bilateral clavicle heads and sternum debridement and wound closure with a left pectoralis major flap to fill the space was performed (Fig. 2D, E). Pathologically, the debrided tissue showed granulomatous inflammation with necrosis, but its cultures were negative. The antibiotics were changed to orally administered linezolid and clofazimine, and she was discharged 53 days after wound closure (total admission period: 317 days). Although swollen lymph nodes remained on CT, no recurrence was observed 5 months after the antibiotics were completed (total sensitive antibiotics use: 366 days).

## Discussion

*M. abscessus* is classified as a rapidly growing mycobacteria (RGM) in non-tuberculosis mycobacteria (NTM). RGM skin and soft tissue infections (SSTI) tend to require a longer duration (average 49 to 83 days) than other general bacteria to present symptoms (such as cellulitis, nodules, and abscess) [3–6]. Moreover, although RGM is identified by acid-fast staining rather than general culture, this process could sometimes be skipped due to underestimation or low recognition of SSTI with acid-fast bacteria because of its infrequence, which could result in delayed definitive diagnosis and treatment initiation. Time from symptom onset to definitive diagnosis has been reported to be 57–85 days [6]. Therefore, RGM infection should be considered in patients with delayed local manifestations of infection.

Among the RGMs, *M. abscessus* infection is challenging to treat because of its high multi-drug resistance. There is currently no standard treatment for *M. abscessus* SSTI, but a combination of antibiotics with macrolide and other intravenous agents such as amikacin, cefoxitin, or imipenem, is generally recommended. Surgical drainage is also indicated for extensively progressive lesions and abscess lesions that are not likely to respond to antibiotics [3, 7]. Moreover, the removal of related foreign bodies or devices should be conducted when feasible [1]. Reportedly, 69.6% of patients are treated with antibiotics and surgery [6].

In the present case, the right upper lobe, which showed inflammatory changes, reminiscent of NTM infection, was resected due to tumor invasion. Although NTM was not evident in the initial bronchial lavage culture, this could account for the presence of M. abscessus. A tendency for NTM infection has been reported in immunosuppressed patients; therefore, we should have paid more attention to this possibility. Moreover, several disseminated lesions in the right axial, parasternal, and bilateral supraclavicular lymph nodes occurred over the treatment period with local sternal drainage and antibiotics therapy. Delayed diagnosis, macrolide-resistant M. abscessus, and interruption of antibiotics therapy could account for the spread of the disseminated lesions [1]. Furthermore, the disseminated lesions were determined to be difficult to remove because of their number and depth; thus, long-term antibiotics therapy with NPWT was chosen, but without favorable results. This may be attributable to the residual sequestrum, which was indicated on short tau inversion recovery image as high-intensity regions [8]. However, the negative culture results of the pus drainage from two disseminated lesions indicated good infection control in the lymphadenitis, suggesting that complete infection control could be achieved with localized procedures. Consequently, no disease progression was observed five months after wound closure. Thus, localized procedures could be an option for patients with controlled disseminated lesions (determined by the results of cultures and blood examination). Therefore, repeatedly performing cultures from disseminated lesions could be an important means of assessing the infectious condition and thereby facilitating the development of therapeutic strategies, including additional localized procedures.

## Conclusions

We presented a successfully treated case of postoperative *M. abscessus* sternal infection with disseminated lymphadenitis. To reduce the period of inefficient treatment, acid-fast staining should be obtained for late-onset infectious manifestations in wounds suspected of RGM infection. Repeated culture assessment of disseminated lesions is recommended to facilitate the development of appropriate therapeutic strategies. Localized procedures may be an option for patients with controlled disseminated lesions evidenced by negative cultures.

## Data Availability

All data generated or analyzed during this study are included in this published article.

## Abbreviations

AMK: Amikacin
CAM: Clarithromycin
IMP/CS: Imipenem/cilastatin
*M. abscessus*: *Mycobacterium abscessus*
NPWT: Negative pressure wound therapy
NTM: Non-tuberculosis Mycobacteria
RGM: Rapidly growing mycobacteria
SSTI: Skin and soft tissue infections
